# Ontology-Enabled Emotional Sentiment Analysis on COVID-19 Pandemic-Related Twitter Streams

**DOI:** 10.3389/fpubh.2021.798905

**Published:** 2021-12-06

**Authors:** Senthil Kumar Narayanasamy, Kathiravan Srinivasan, Saeed Mian Qaisar, Chuan-Yu Chang

**Affiliations:** ^1^School of Information Technology and Engineering, Vellore Institute of Technology, Vellore, India; ^2^School of Computer Science and Engineering, Vellore Institute of Technology, Vellore, India; ^3^Electrical and Computer Engineering Department, Effat University, Jeddah, Saudi Arabia; ^4^Department of Computer Science and Information Engineering, National Yunlin University of Science and Technology, Yunlin, Taiwan; ^5^Service Systems Technology Center, Industrial Technology Research Institute, Hsinchu, Taiwan

**Keywords:** sentiment analysis, emotion ontology, natural language processing, twitter streams, latent Dirichlet allocation, SPARQL

## Abstract

The exponential growth of social media users has changed the dynamics of retrieving the potential information from user-generated content and transformed the paradigm of information-retrieval mechanism with the novel developments on the concept of “web of data”. In this regard, our proposed Ontology-Based Sentiment Analysis provides two novel approaches: First, the emotion extraction on tweets related to COVID-19 is carried out by a well-formed taxonomy that comprises possible emotional concepts with fine-grained properties and polarized values. Second, the potential entities present in the tweet can be analyzed for semantic associativity. The extraction of emotions can be performed in two cases: (i) words directly associated with the emotional concepts present in the taxonomy and (ii) words indirectly present in the emotional concepts. Though the latter case is very challenging in processing the tweets to find the hidden patterns and extract the meaningful facts associated with it, our proposed work is able to extract and detect almost 81% of true positives and considerably able to detect the false negatives. Finally, the proposed approach's superior performance is witnessed from its comparison with other peer-level approaches.

## Introduction

Emotion Analysis helps us to understand and to retrieve the potential emotional information from any user-generated content. The user-generated content can be text, images, animations, videos, scan images, etc. But the text is predominantly used due to the emergence of social media platforms and other sources as well. Extracting emotions from the text document is a challenging task as it covers a wide range of ambiguities and anomalies that persist over the text content. Emotion Analysis has been categorized into different models, such as the corpus-based model, appraisal-based model, and knowledge-based model. The lexical affinity of a word or a phrase is determined based on the probability of emotion attached to each term and the cognitive factor will be inclusively considered for evaluation. For example, the term, “eager” takes a high probability score against “curious” than “willing.” To shun these difficulties, an ontology-based analysis has been used predominantly to disambiguate the terms and to yield a high precision score for the emotional terms.

At first, the term, ontology was inhibited from psychology but it started making its footprint in computer science from the year 1980s. Ontology-based emotion extraction on text documents has been a research focus for many applications and it has gained huge momentum in recent years (1–3). As human beings always use natural language to represent the domain of the specific text document, the ontologies have been using formal language representations to describe the domains of the input document. The use of ontologies has been widely applied in many research areas, such as artificial intelligence, entity extraction, Semantic Web, collaborative software development, and many more (4, 5). Ontologies provide huge benefits, such as conceptualization, reusability, sharing of the resources, and coreferencing the terms. It is indeed a cost-effective process for dealing with text analysis and is very robust for many application areas as mentioned above. Many research works have been implemented with ontologies to remove the ambiguity that persists in the textual content. A structure that provides a formal description of a standard representation of real-world concepts can improve the understanding of these concepts. COVID-19 is the disease caused by SARS-CoV-2, the coronavirus that emerged in December 2019 (6–11). The lack of existing studies allows us to explore this ontology-based emotion extraction on COVID-19 datasets.

The conventional Sentiment Analysis is normally applied to the text to determine whether the given text expresses a positive sentiment or a negative sentiment. Generally, the Sentiment Analysis has been carried out for knowing the feedback or the opinion of the customers about the products they had purchased. In particular, the following two Sentiment Analysis approaches were usually performed to get the sentiment score of the text documents: lexicon-based approach and machine learning-based approach. The first approach segments the text into appropriate morphological lexicons and then extracts the opinion words which are usually expressed either positive or negative. A dedicated dictionary that has been established for opinion words is called a lexicon dictionary and mapping of the opinion words on the lexicon-based approach has been done based on this lexicon dictionary. But in this case, tweets are not considered because of the underlying fact that tweets are within 140/280 characters and normally, words in a tweet are condensed into canonical forms due to their limited length. The machine learning-based approach has now gained huge momentum for many research activities and in that case, a specialized sentiment classifier has been trained for the textual corpus to predict the polarity of the text. Typically, the sentiment classifier has trained the model based on bigram or n-gram textual representation and classifies the text into the respective sentiment scores. The major drawback of this approach is the manual labeling of the training datasets and it has not been worked out well for tweets or any short messages.

The ontology-based approach proposed by Ali et al. (12) indicates that the sentiment score has not been made possible based on the emotional polarity but on the accuracy score obtained from the ontology. The ontology-based model helps to extract the emotions from the tweets based on the factors, such as concepts, the relationship between the concepts, characteristics of individual concepts, and external source document support for disambiguation. In this connection, Semantic Web technologies have been used to construct the ontology for extracting the emotions from the text documents and allow for sharing and reusing of the potential data for various applications. Resource description framework (RDF) is used to identify the resources by Uniform Resource Identifier (URI), and Resource description framework schema (RDFS) helps to organize and formulate the content in the machine-understandable format. The SPARQL query is used to fetch the disambiguous results from the RDFS graph. Therefore, we proposed the novel approach to segment the tweets into appropriate morphological textual representations and to train the classifier to distinguish the polarity of tweets using Semantic Web technologies and natural language processing (NLP) toolkits.

The primary objective of this research work is to quantify the efficient classification of the extracted emotional entities from tweets using the proposed emotional ontology. Following are the outlined objectives of the proposed work:

- The domain entities have been classified with appropriate upper ontology classes and it measures the correctness concerning the metrics given in the Emotion Ontology.
- Selecting and quantifying some of the general domain emotional entities from the tweets that have significant relevance in the upper ontological class of Emotion Ontology.
- Determining the word embedding and taxonomy-based similarity measures for the extracted emotional entities.
- Generating relevant descriptions for those extracted emotional entities and assigning the relevance score based on the results obtained from the SPARQL query.

The rest of the paper is organized as follows: Section Related Works summarizes the existing works based on semantic similarity measures, ontological features, emotion analysis, and sentiment analysis of social media content. Section Proposed Approach for Ontology Creation highlights the important features of Domain Ontology and semantic enrichment of entity categorization. Section Utilization of Emotion Ontology describes the core aspects of designing the semantic-based emotion ontology and taxonomy-based entity classification and disambiguation. Besides, it also explains the importance of ontology modularization for partitioning the large-scale ontologies into some self-contained modules. Section Conclusion delineates the polarity calculation and determines the performance of emotion ontology for the fine-grained measures.

## Related Works

Extracting emotions from the documents is a tedious task as it involves a wide range of subjects, such as psychology, anthropology, society, and biology. In psychology, emotion is defined as the study of different orientations of human beings and the theories of emotions have been represented using cognitive psychology. Even though many cognitive models had been evolved to denote the emotions incurred in the documents, the most widely implemented affective computing models are dimensional (13), categorical, (14), and appraisal (15). Lang (13) has extensively studied and analyzed the expressions of emotions for effective detection and categorization. He had classified the emotions into three vital categories, namely, subjective emotions, behavioral emotions, and psychophysiological emotions. The subjective emotions were classified based on perceived emotions vented by the user. The behavioral emotions were distinguished by facial, gestural, and speech paralinguistic attributes. The psychophysiological emotions were identified by the heart rate, electroencephalographic results, galvanic skin values, etc.

Later, Lacy (16) had delineated the behavioral models with emotional processes and the methods required to indicate the various stages of emotions are represented by cognitive psychology. They further analyzed the emotional processes very deeply and came out with three critical parameters, to access the behavioral model, such as emotion itself, emotional context, and multimodal behaviors. The study added that time-of-event is a critical factor for representing the emotional context. On the contrary, Baldauf et al. (17) had proposed a novel model in which they assessed the behavioral model in terms of location, time, the person involved, the state of the person at the time of the event, societal activity, and instruments or devices used. These parameters were used for emotional context and augmented the emotional credibility for research analysis.

Later et al. (18) have extended the sentiment analysis of Twitter streams by computational frameworks and analyzed the emotions expressed in Twitter streams by certain questionnaires. The answers pertaining to the questionnaires were later analyzed by the popular probabilistic topic model, latent Dirichlet allocation (LDA) algorithms that effectively disambiguated the ambiguous emotions present in the tweets. Their work highlighted the various factors of emotion discovery shifted among various users' conversations on Twitter.

The emergency of social media has created a huge opportunity for social users to interact and discuss a wide range of topics prevalently happening around the world. These discussions paved the way for analyzing the significant interest of the social media users over the topics and performing sentiment analysis, such as emotion detection and recent opinion mining. According to Ren and Hong (19), emotion analysis had been widely implied in various domains, such as e-mail content, novels, online news content, blogs, dashboards, and other social media content. Researchers, such as Oliveira et al. (20) and Ren et al. (21) have studied the consumers' views on many online products and their purchases and later predict the growth of stock markets. They forecasted the growth of stock markets based on discussions that happen on Twitter and followed the lexical heuristic approach to filter out the basic emotional values presented in the tweets.

The researchers, Baldoni et al. (22) and Dey et al. (23) have implemented the emotion extraction process first by n-ary relations. The complex relations were factorized into some set of binary relations and the potential named entities were extracted from the text. Extracting potential named entities from the text is a complex task and it requires a well-equipped classifier to identify and distinguish the potential entities from the text. So, the classifier has been well trained to suit the needs of the entity extraction and in this case, the learning approach techniques have been used and in particular, the maximum entropy model has been followed for entity extraction. Once the potential named entities have been identified, the complex relationship between the entities has been reconstructed using a simple directed graph. The three significant approaches followed before the emotional analysis processes include (i) rule-based approach, (ii) learning approach (maximum entropy), and (iii) graph-based approach.

Earlier, open information extraction (OIE) has gained huge momentum in extracting the entities and relation sets from the documents. OIE is a full-fledged extraction framework Daniel et al. (24) for both structured and semi-structured documents. OIE enables the extraction process in place of DBpedia and LinkedGeo data for effective entity detection and categorization. Further, it paves way for entity triple formation using the Semantic Web technologies, such as RDF/RDFS and Web Ontology Language (OWL).

Most of the semantic similarity measures attempt to emulate the human ability to evaluate the level of relativeness between the words according to their semantic evidence. According to Madani et al. (25), semantic measures assess the quality of the semantic associations between the words, as indicated by the investigation of semantic proxies (words, senses). For instance, a semantic similarity measure would not think about the two ideas “sloth” and “monkey” to be comparative, regardless of whether the vast majority think sloths to be monkeys. Given that semantic estimates target contrasting things concurring with their significance caught from semantic proof Mozafari et al. (26), it is hard to additionally characterize the thought of semantic measures without characterizing the ideas of meaning and semantics. Likewise, the two ideas, “tea” and “cup” are in this way exceptionally related despite the way that they are not comparable: the idea, “tea” alludes to a drink, and the idea, “cup” alludes to a vessel. In this manner, the two ideas share not many of their constitutive properties. This features a potential understanding of the idea of semantic similarity, which can be comprehended in terms of replacement, i.e., assessing the suggestion to substitute the analyzed components: Tea by Coffee or Tea by Cup. In fact, word-to-word semantic similarity is sometimes assessed not just considering (close) synonymy, or the lexical relations which can be considered as comparable to the ordered connections for words, e.g., hyponymy and hypernym.

Table 1 highlights the performances laid out on semantic similarity measures of different domains.

**Table 1:** Highlights of semantic similarity measures and dataset assignments.

**S.No**	**Model references**	**Year**	**Core evaluation method**	**Data size**	**Inter-agreement**
1	Dridi and Recupero (27)	(2019)	Semantic similarity Association between the pairs of nouns	40	⋎ = 0.784
2	Mozafari and Tahayori (26)	(2019)	Semantic relatedness exists among the pairs of nouns.	Two sets 150/200	ρ = 0.661
3	Dragoni et al. (28)	(2018)	Semantic proximity of word pairs on the basis of synonymy questions	50	NA
4	Chen et al. (29)	(2017)	Semantic similarity evaluation of words based on analogy questions	390	NA
5	Li et al. (30)	(2017)	Semantic Associativity between 25 medical words	34	⋎ = 0.51
6	Zhu and Iglesias (31)	(2016)	Semantic similarity and proximity score of pairs of UMLS concepts (domain: medical)	571	*r* = 0.50 (sim) *r* = 0.47 (rel)
7	Bruni et al. (32)	(2014)	Semantic relatedness of pairs of words	2500	ρ = 0.74
8	Hill et al. (33)		Semantic similarity of pairs of words	999	ρ = 0.7

## Proposed Approach for Ontology Creation

**Figure 1:**
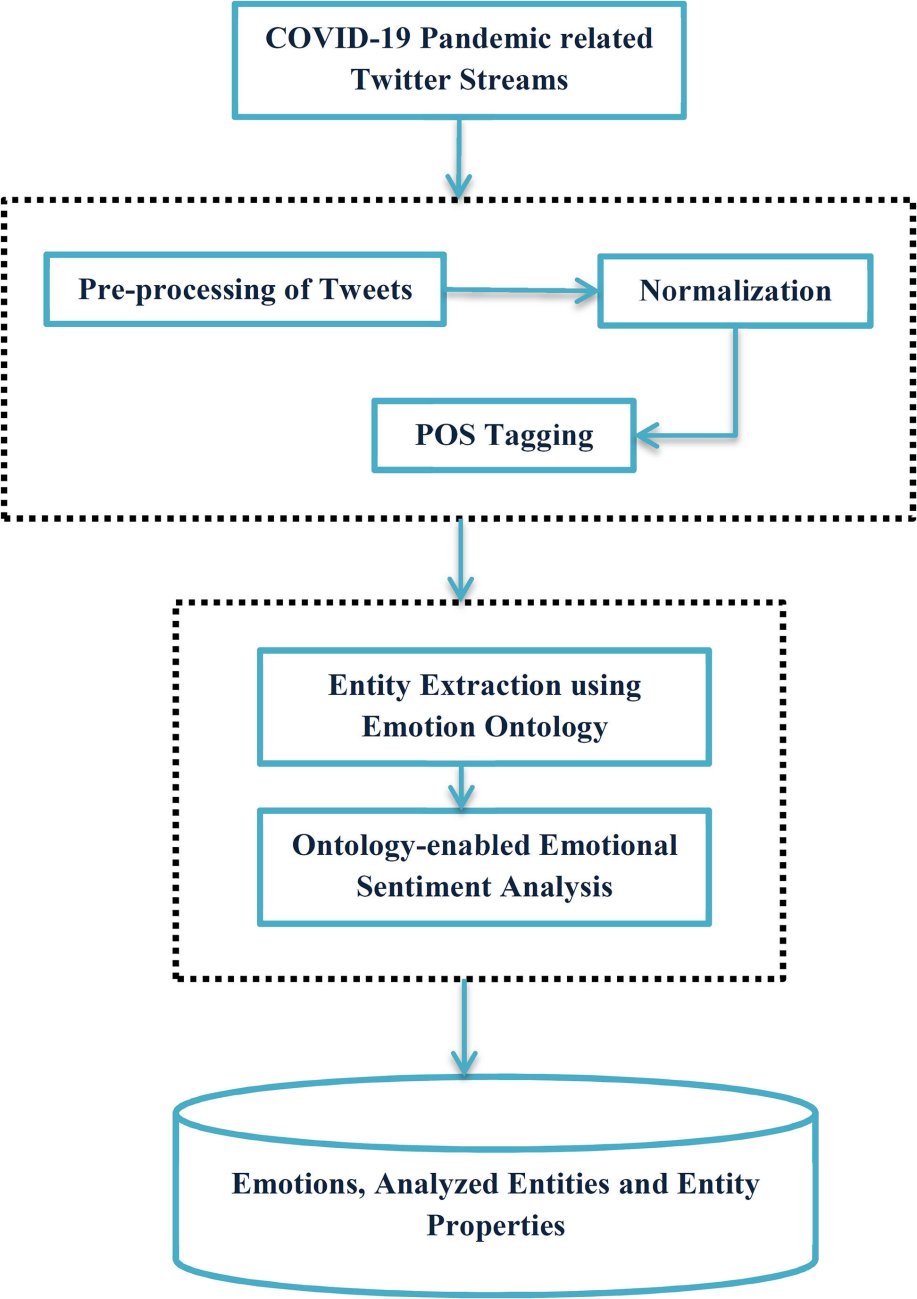
Proposed architecture for emotion extraction from Tweets.

At first, the proposed approach deals with the creation of Domain Ontology for the bio-medical-related subjects to provide an accurate sentiment score for the opinion words present in the COVID-19 tweets. Here, the use of Semantic Web technologies gains a tremendous prospect to create Domain Ontology for the specific subjects, and further, the opinion words from the subject have been enlisted for the ontology with appropriate properties and literals. The Domain Ontology plays a seminal role in supplementing the feature extraction process to yield the precise sentiment score for the COVID-19-related tweets and increase the accuracy rate of the results. The proposed system is divided into two crucial phases. (i) Creation of Domain Ontology for the selective subject and (ii) feature extraction of opinion words included in the Domain Ontology. Figure 1 illustrates the proposed architecture for emotions extracted from the tweets.

### Creating the Domain Ontology

Normally, the Domain Ontology can be created either by utilizing the existing off-the-shelf ontologies or developing the ontologies based on the suitable requirements. There were many off-the-shelf ontology resources available for specific domains in the Semantic Web Forums that paved a way for the researchers to utilize these resources to map the properties of the ontology and opinion words (34). Many software solutions have been completely dedicated for knowledge-based entity extraction and deployed further with the extraction of context-specific ontology/structured terminologies, such as the disease ontology (DO), unified medical language system (UMLS), WordNet, Medical Subject Headings (MeSH), Gene Ontology, etc. To support the OWL, the standard RDF grammar has been designed with stipulated functionality (31). These ontological frameworks (28) can be used to define the terms and entities generically. In Table 2, we have given the name of the tools used for ontological design, supporting formats, libraries, command-line interface (CLI), and source code library (LIB). As the ontological frameworks support the programming languages, such as JAVA and PYTHON, the semantic similarity measure can be taken into consideration for some pair of concepts (P) or some association between the groups of concepts (G).

**Table 2:** Semantic tools and ontology design for CLI and LIB.

**Name**	**Ontology**	**Types**	**Measures**	**Language**
SML	OWL,RDF, and OBO	CLI, LIB	P, G	Java
FastSemSim	OBO and others	CLI, LIB	P	Python
OntoSim	OWL, RDF	LIB	P	Java
YtexSemanticSimilarity	SEE DOC	LIB	P, G	Java
SimilarityLibrary	Wordnet, MeSH, GO	CLI, LIB	P, G	Java / Python
OWLSim	OWL, RDF, OBO	LIB	P	Java / Python

### Formal Concept Analysis for Ontology Creation

Formal concept analysis (FCA) is a mathematical model that has been mostly applied in knowledge representation and information extraction. The sole objective of FCA is to pave the way for creating the Domain Ontology by a step-by-step approach and by considering all the ambiguities that persist on the properties of the Domain Ontology. With the advent of recent Semantic Web Technologies, the OWL helps to derive the potential objects and their properties and establish the Domain Ontology for accurate knowledge representation.

#### Basic Elements of FCA in Domain Ontology

The fundamental building block in FCA is the concept that can be derived from two valuable sets: Extension and Intention. The Extension is a set of objects collected for the Domain Ontology in considering the potential opinion words listed for the specific subjects. The set of objects has been chosen based on some grounding rules formulated for creating the ontology. The Intention is a set of attributes for every selected object and each attribute has some inherent properties and literals to represent in the hierarchy of domain levels. Every object that represents the concept has its attributes in the intention and likewise, each attribute linked to the concept of the Domain Ontology can be further shared with all the other objects of the Extension. This mutual connection that exists between the objects and attributes is described through the mathematical representation called Formal Context. A Formal Context is a triple that can be represented as K (O, A, I). Here O is a collection of objects for the Domain Ontology, A is the collection of potential attributes of the objects, and I is a binary correlation that exists between the objects and attributes; I ⊆ O × A, where (o, a) ∈ I (i.e., “object o has to attribute a”).

### Method of Ontology Learning

Ontology Learning or ontology acquisition is the inbuilt process of automatically inheriting the concepts and their associated properties from the other ontology resources or datasets (1). This capability has helped the researchers to save time and customize their Domain Ontology as per their requirements. In this study, we have used OntoGen, a semi-automatic ontology editor that reduces the overall deploying time of the ontology, as well as the complexity, which persists over the hierarchy of the ontology. In nutshell, this editor helps resolve the conflict between the ontology editors and domain experts because both do not hold the required ontology-engineering skills. That is, OntoGen is an interactive editor that can help the domain experts to accept or reject the concepts or objects, or properties of the ontology, based on their requirements, and suggests the appropriate concepts and relations to the ontology. The manual adjustments of assigning instances to the concepts can be done at any level of the ontology hierarchy and thereby reduce the complexity of the overall ontology representation.

### Semantics Entailment

The domain ontology has been created *via* FCA and Ontology Learning and it enriched the taxonomy of a set of concepts and its potential attributes. To boast the semantic representation of the concepts, the ontology has now been filled with appropriate synonyms and hyponyms of the corresponding attributes. For instance, the term, “apple” comes under the class, “fruit” as well as “phone”; further, their hierarchy extends above to “vegetables” for “fruit” and “electronics” for “phone.” The synonyms and hyponyms can be fetched through the well-formed lexical corpus called WordNet (35, 36). With the assistance of WordNet, we extracted the appropriate synsets for the word and appended the same to the ontology and associated with the appropriate attributes of the object. We have been using the Semantic Web Language for the ontology called OWL which has the following three dedicated categories: OWL DL, OWL Lite, and OWL Full (16). To augment the synsets extracted from WordNet to the Domain Ontology, we preferred to use OWL DL which has the syntax owl: subPropertyOf and owlequivalentProperty. Through these OWL properties, we updated the synonyms and hyponyms, respectively, into the ontology.

### Sentiment Analysis on Twitter Streams

After the creation of domain ontology, the next phase of the operation largely relies on performing the Sentiment Analysis of the tweets and distinguishing the polarity of the extracted opinion words from the tweets. The whole process constitutes of filtering the set of tweets from the COVID-19 datasets and then extracts the potential opinion words from the tweets and maps the opinion words in the domain ontology for getting the sentiment score (37, 38). To obtain the sentiment score of the opinion words, we have scaled up three distinct procedures: (i) querying the Domain Ontology for retrieving the correct attributes of the object, (ii) distinguishing the ambiguities that persist over two or more objects, and (iii) disambiguating the objects with necessary attributes and literals.

#### Step#1: Utilization of the Ontology

The Domain Ontology has been used to extract the appropriate attributes for the opinion words and to fetch equivalent classes for the objects with necessary attribute references. This task can be performed through Jena Fuseki, which is a Java API for handling RDF/s and OWL codes (39). Generally, JENA API is used to filter the results in the form of a triplet which is represented as Subject, Predicate, and Object (SPO). This triplet is used to discriminate the classes of the ambiguous attributes and return the results either in the form of an RDF Graph or as JSON format. Once we obtain this sort of ontology-based hierarchy model for classes and attributes, retrieving the triplet would be easier and this, in turn, results in matching the opinion words in the Domain Ontology.

#### Step #2: Identify the Ambiguous Entities in the Tweet

In the preprocessing steps of tweet normalization, we removed all the special symbols like @, #, and other uniform resource locators (URLs), and then considered the words which are deemed to be opinion words after verifying them with the opinion-lexical database. The pre-processed tweets are then added to the second phase of the Sentiment Analysis and here we matched the opinion words which are also called entities in the Domain Ontology. The mutual mappings between the opinion words in tweets and their equivalent attributes in the Domain Ontology pose an ambiguity and create a separate list of ambiguous entities for further disambiguation. For logics and inferences possessed in the Ontology, we have used the first-order logics as well as description logics to express the relations given in the Emotion Ontology.

**Table:** Algorithm: 1 Emotional Ontology-Based Entity Extraction and Weight Calculation.

*Input: Parse Tweets and Extract Named Entities NE = (e_1_, e_2_,…..e_n_)*
*Output: Collection of entities mapping with emotion ontology*
*Begin*
* For every entity e_i_ in NE do*
* For every domain D_j_ in EmoOnto do*
* if the term e_i_ present in EmoOnto then*
* Add e_i_ into ResultSet R*
* end if*
*For every entity e_i_ ∈ ResultSet R do*
* Calculate the semantic weight sw of term e_i_ as,*
wt = [Σ (Count of e_i_ in d_j_) ] ^*^ [log(n/df)]
*End for*
*End for*
*End for*
*End*

#### Step #3: Disambiguate the Objects

The ambiguous list of entities is then processed for entity disambiguation which is the crucial task of this research work. The core idea behind the disambiguation process is that the mapping between two objects with dissimilar attributes in the Domain Ontology is identified first and then a comparison of properties and literals of two objects can be measured using semantic similarity-based algorithms i.e., Word2Vec and disambiguate the entities which hold the highest similarity score. The fundamental principle of ontology lies in disambiguation so that the original sense of the attributes can be obtained only at the leaf nodes of the hierarchy and hence the semantic similarity measures, such as Word2Vec have been selected to delve deep into the hidden layers of the neural network to get the precise results for the target words. Algorithm 1 illustrates the Emotional Ontology-Based Entity Extraction and Weight Calculation process.

## Utilization of Emotion Ontology

According to Li et al. (40), ontologies are originally stated as a formal, explicit specification of a shared conceptualization. It originally deals with knowledge representation in the hierarchy of concepts ordered in the ontology and it shared the common attributes among other classes in the hierarchy to represent the types, relations, properties, and other interrelation concepts that lie in the ontology. Ontology plays a crucial role in the knowledge extraction process and represents some conventional extraction models, such as keyword-based extraction, bag-of-word models, and other statistical analysis that failed to yield accurate results. Ontologies help share the common attributes and they are made machine-readable. Ontologies play a pivotal role in establishing the mutual connection between class hierarchies and other concepts underlying the objects. Further, the ontologies are very useful for exact labeling and categorizing the mutual relationship that exists between the objects and their properties. The ontologies are defined in the following four forms: (1) an entity that links the object, (2) the relation between the entity, (3) object relationship, and (4) properties that connect the objects. The use of ontology is now gaining momentum and the following are some potential reasons for building the ontology for the given requirements: Examining domain-specific knowledge, distinguishing the domain suppositions, reusing the domain knowledge for diverse applications, converging the domain knowledge and functional designs, and sharing the knowledge with other software bots.

### Emotion Ontology Corpus Creation

**Figure 2:**
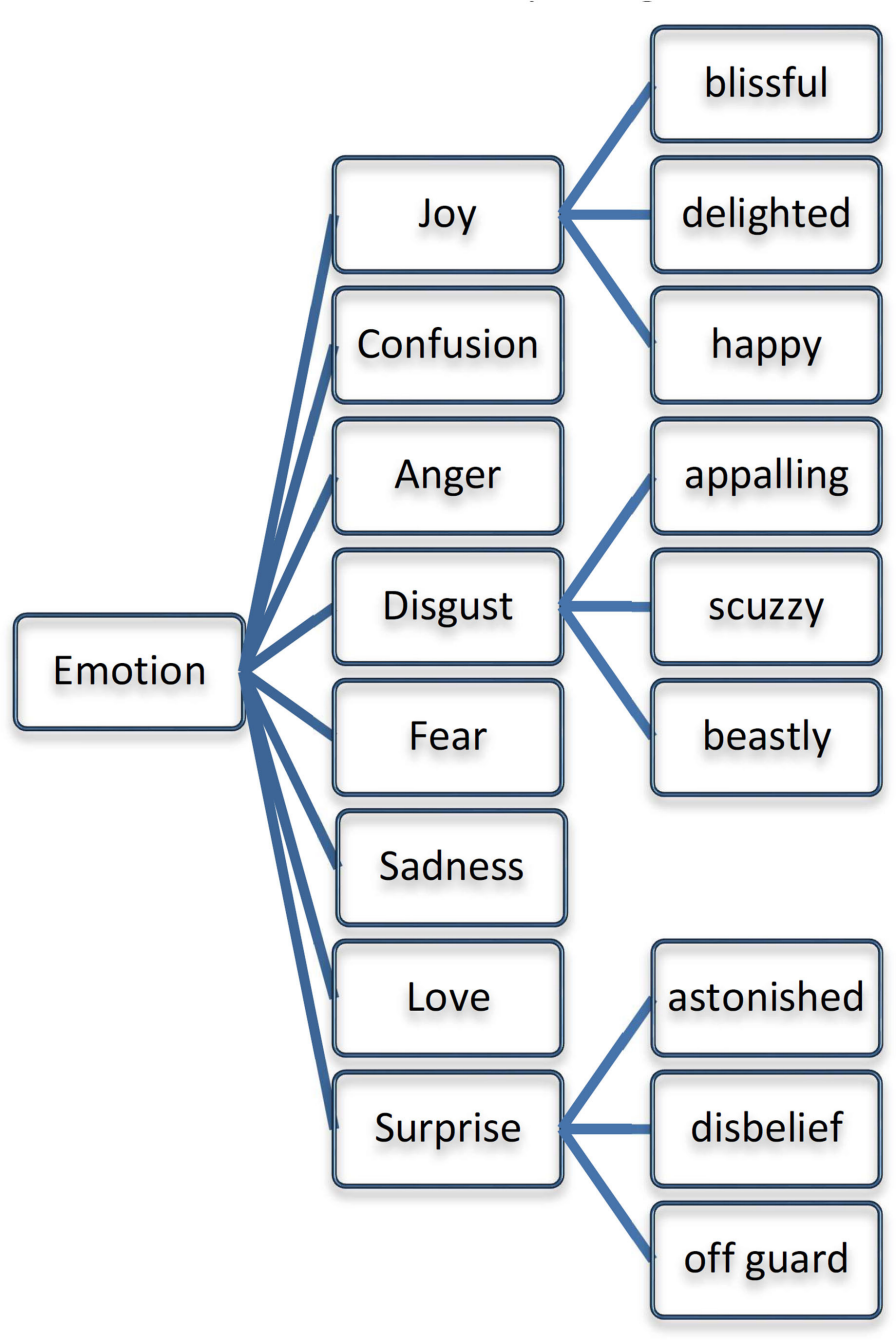
Fundamental emotions list for ontology population.

Eight emotions were selected based on the recommendation given by Ekman (14) and observed that these eight emotions have been widely used in the COVID-19 datasets. We have populated these eight emotions into the Emotion Ontology and represented them as OWL Class (41). A dedicated list of emotional categories of terms has also been chosen based on the psychological models (42) and we structured them in the Emotion Ontology as given in Figure 2. Further, 14 subsets of emotions are added as subclasses for the above eight emotions. Besides, the emotions can be further classified into four types: *Basic Emotions* (joy, confusion, anger, disgust, fear, sadness, love, and surprise), *Mid Emotions* (distraction, boredom, acceptance, apprehension, interest, serenity, pensiveness, and annoyance), *Intense Emotions* (ecstasy, amazement, vigilance, grief, admiration, loathing, rage, and terror) and *Complex Emotions* (disproval, love, submission, optimism, awe, contempt, aggressiveness, and remorse). The Emotion Ontology helps to reduce the complexity of the redundant emotion population and also paved the way for detecting the emotions represented in the tweets. Besides, Ontology minimizes the non-emotional terms, which would be very large in Twitter Streams.

**Figure 3:**
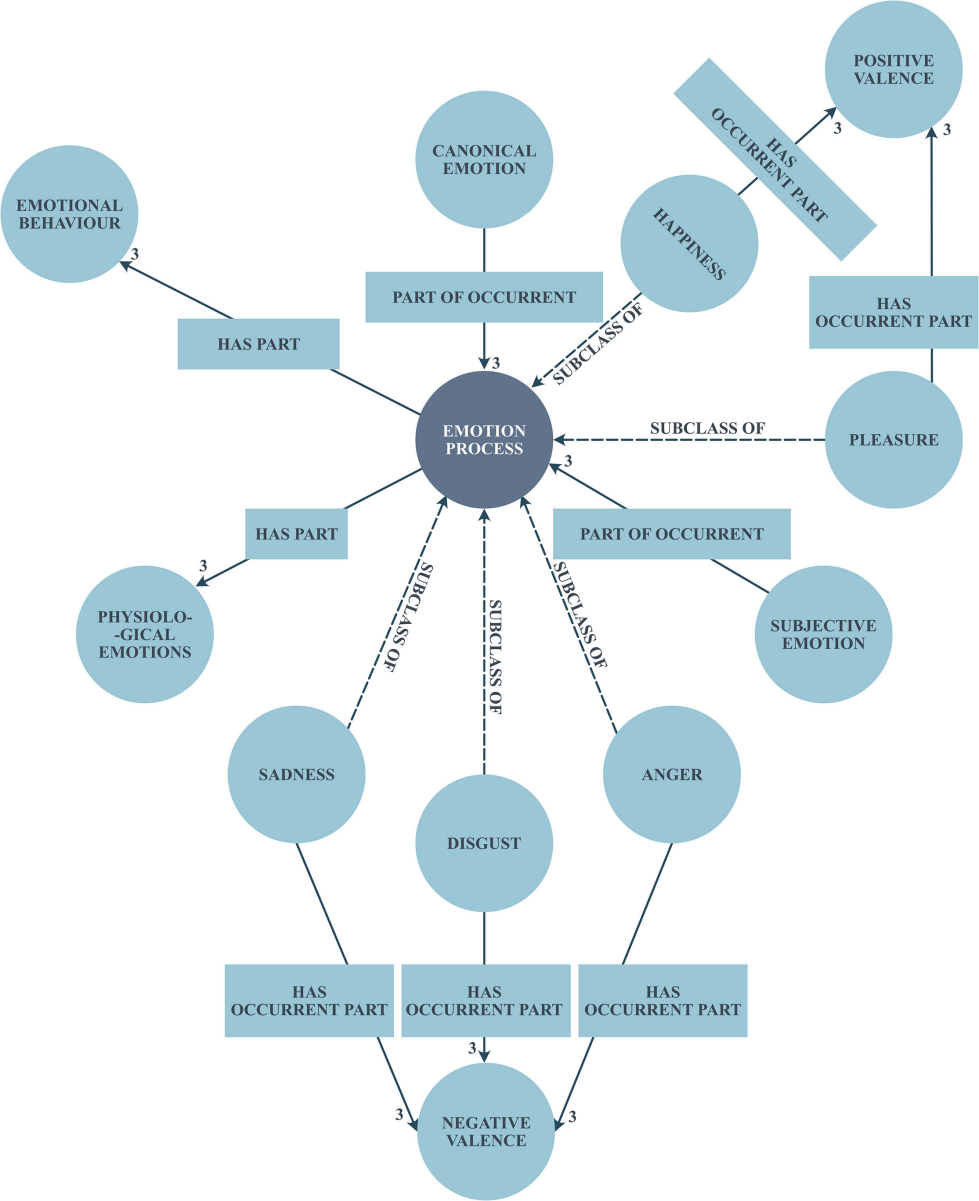
Emotion ontology generation.

The Domain Ontology has been created manually with the help of a psychological-emotional words list and WordNet. But when it comes to the reusability of several classes belonging to different domains, the manual population of ontology would be a hard task. Further, it would lead to duplication of many more properties and their values. Therefore, effective utilization of Semantic Web technologies, such as RDF/RDFS, SPARQL, and OWL would primarily facilitate the mutual connection that exists over other domain ontologies, such as the one given in the Linked Open Data (43). In this proposed approach, Emotion Ontology has been created based on the effective reuse of existing ontologies and with well-formed semantic vocabularies, such as a friend of a friend (FOAF), semantically interlinked online communities (SIOC), Dublin core, etc. The SPARQL query has been generated to map the semantic vocabularies and other domain ontologies appropriately. Figure 3 illustrates the generation of Emotion Ontology.

### Retrieving and Annotating the Twitter Streams

In recent times, machine learning techniques have been used to automatically annotate the text documents and to train the classifiers according to the needs of the ontological requirements and domain hierarchy (2, 4, 29). But the machine learning techniques are not suitable for unstructured documents and they further lead to many serious implications, such as morphological change, lexical error, sense overlap, and ambiguous annotation for entities. Hence, we proposed a novel approach called ontology-based entity extraction and constructed a novel Emotion Ontology. As the Twitter Streams contain diverse information from different sources, the distribution of emotions that persisted on these tweets is deemed essential for extracting more emotions which further helps in tracking the implicit emotions of individual users on Twitter.

To gain a high recall score for the emotion extraction from the tweets, we have also crawled for slang words and out of vocabulary (OOV) words present in the tweet. The slang words and OOV words were disambiguated and we generated the correct term for the slang words using online slang dictionaries, such as NoSlang Dictionary, Urban Dictionary, and Translit (44). Further, for every detected slang word, we generated the candidate lists based on the synsets produced from the WordNet Synset. We then used Dictionary.com, Thesaurus.com, and Oxford Dictionary for increasing the probability strength of the words. The intensity of the words resulting from these dictionaries would be considered and populated for our Emotion Ontology.

### Emotion Detection in COVID-19 Datasets

Here, we provide a baseline approach that can be followed for automatically annotating the emotions extracted from the COVID-19 tweets^1^. To extract the emotions, we have applied the binary Support Vector Machine (SVM) classifier to effectively identify the eight emotions as proposed by Ekman (14). Each classifier has been trained independently for every emotion which resulted in seven independent binary classifiers and further the merger of these combinations of classifiers would be considered as a single multi-label classifier. Using this single multi-label classifier, the tweets can be annotated even if they possess more than one emotion and return positive if the multi-binary classifier is applied to the tweet. Likewise, it would return negative results if a binary classifier is applied. The result would be neutral for no emotions in the tweet. Each classifier has been following an independent classifier for a different group of features and in particular, the feature used by the binary classifier is a subset of the features deployed in the multi-label classifier (45).

To augment the feature extraction process accurately, we have omitted some of the similarity features which envisioned more on a topic than emotions and rejected the synsets followed by WordNet Affect features in this research. The decision has been taken not to use WordNet Affect for this research as normally the tweet does not possess sufficient synsets to cross-verify over the WordNet Affect. Moreover, WordNet Affect would probably look for possible candidate hypernyms instead of potential resources (i.e., thing, or object, in our case). Another dissimilarity observed on WordNet Affect is that it focused mainly on bigrams and trigrams for normalization instead of phrases. This would not be largely amicable for tweets due to their constraint in length. However, for the effective utilization of feature extraction for the proposed research, we used the topic modeling approach to discover the hidden terms and phrases between the tweets even though the tweet has no entities in common. Here, we have employed the LDA algorithm for topic modeling and it discovers the similarity that exists among the collections of tweets (i.e., the collection of tweets can be considered as a document by the LDA algorithm). For implementing this approach, we have enforced MALLET-based LDA implementation and treated every tweet as its document (46). This approach would project the probabilistic combination of words for every tweet and each topic is generalized into a set of probabilistic combinations of words.

### Tweet Polarity Calculation Mechanism

The ultimate objective of this proposed approach is to calculate the polarity of every tweet that we have collected from COVID-19 datasets. In the conventional methods, the sentiment score will be calculated based on the word polarity and the overall strength will be estimated on the grounds of the polarity score obtained from the probability of the positive and negative words. But in this proposed approach, we have modeled a novel approach to get the polarity of words on every tweet by using the SPARQL query that crawls through the emotional ontology to fetch the correct sense for the search word. In our proposed model, we have excluded the pronouns, adjectives, and articles from the tweets and extracted the tokens such as nouns, verbs, objects, and determiners. As the Semantic Web gives a well-defined meaning to the sentences and converts the sentences into appropriate triples, such as SPO, we have followed the same pattern for our proposed approach for the Emotion Ontology building process and extracted the nouns (subjects), verbs (predicates), and objects (objects) that satisfy the Semantic Web triple pattern, respectively. The relation between the entities (either noun or object) can be expressed using the RDF/RDFS. The ontology population can be done either by the tool Protégé or using the Semantic Web language, OWL. For logics and inferences possessed in the ontology, we have used the first-order logics as well as description logics to express the relations given in the Emotion Ontology. Mostly, we have utilized the predicates for relationships between two or more entities and the predicate relationships can be expressed using the OWL predicate type method. For each tweet, we execute the SPARQL query to obtain the disambiguated result. The SPARQL query to fetch the entities embedded with emotions is given below:The Emotion Ontology helps to disambiguate the search terms accurately and to return the results precisely. Thereby, the polarity and sentiment score for each term or entity can be obtained and yield an accurate sentiment score for the tweets. Suppose, the search terms used for the execution in the SPARQL query return NIL results, then the context of the token would be considered and an appropriate parameterized SPARQL query will be executed against the Emotion Ontology to fetch the right mix of words for consideration. We then estimated the sentiment score by considering the largest domain value from the list of positive tokens and the smallest values from the other set of negative tokens. Eventually, to determine the polarity of every tweet, the tweet sentiment score is calculated based on the strength score of the tweet, and the strength score of every entity in the tweet is calculated based on the following calculations: (a) positive, if sentiscore is greater than 1, (b) negative if sentiscore is less than or equal to −1 and (c) otherwise neutral.

**Table:** 

SELECT ?group ?emotion
WHERE
{
?a rdf:type x:EmotionAboutEntityItem;
x:hasEmotion ?emotion;
x:hasEntityItem ?entityItem.
?group x:contains ?entityItem.
}
ORDER BY ASC(?group)

Further, to assess the absolute performance of our proposed Emotion Ontology, we have considered two implicit functions, such as sklearn.metrics.recall and sklearn.metrics.f1 to calculate the potential values, such as recall and F1 score, respectively. These two metrics were used to handle the multi-label classification (positive, negative, and neutral). Our sheer Emotion Ontology population using Semantic Web technologies and performance measures selected for sentiment score has enabled the proposed method to avoid manual annotation using any machine learning techniques, and the problem of manual annotation has been resolved. Further, many recent studies have sought the help of machine learning algorithms to manually annotate the entities given in the text or any tweets. The proposed Emotion Ontology can deal with dynamic domain vocabularies that would possess different representations in the text.

### Experimental Evaluation of Emotion Ontology Matching

Table 3 shows the performance of our Emotion Ontology and categorizes the accuracy rate based on the different segmentation followed for tweet analysis. Recall of the proposed ontology pinpoints the various expression and candidate terms employed over the tweets and reduced the false-positive cases encountered. The tweet segmentation has been carried out for tweets, part-of-speech (POS) tagging, and the likelihood of context rule applied for the analysis.

**Table 3:** Tweet segmentation and classification for ontology matching.

**Segmentation**	**Precision**	**Recall**	**F-Score**
Tweet Seg + Ontology	0.892	0.801	0.841
Tweet Seg + Inferences	0.761	0.684	0.692
POS + First Order Logics	0.561	0.504	0.539
Tweet Seg + POS	0.710	0.693	0.703

From the COVID-19 datasets retrieved from Twitter, training was strenuously performed over all the sample tweets, and validation was carried out with the baseline gold standard datasets. The total number of occurrences for each emotion listed was calculated by the number of positive values on the respective emotions and identified from class imbalances over the emotions, such as joy and love. Table 4 outlines the occurrences of each emotion tested for the COVID-19 dataset.

**Table 4:** Total number of occurrences of the emotions tested for the training dataset.

**Joy**	**Anger**	**Disgust**	**Fear**	**Surprise**	**Love**	**Sadness**	**Confusion**
187	562	491	688	490	294	579	463

**Figure 4:**
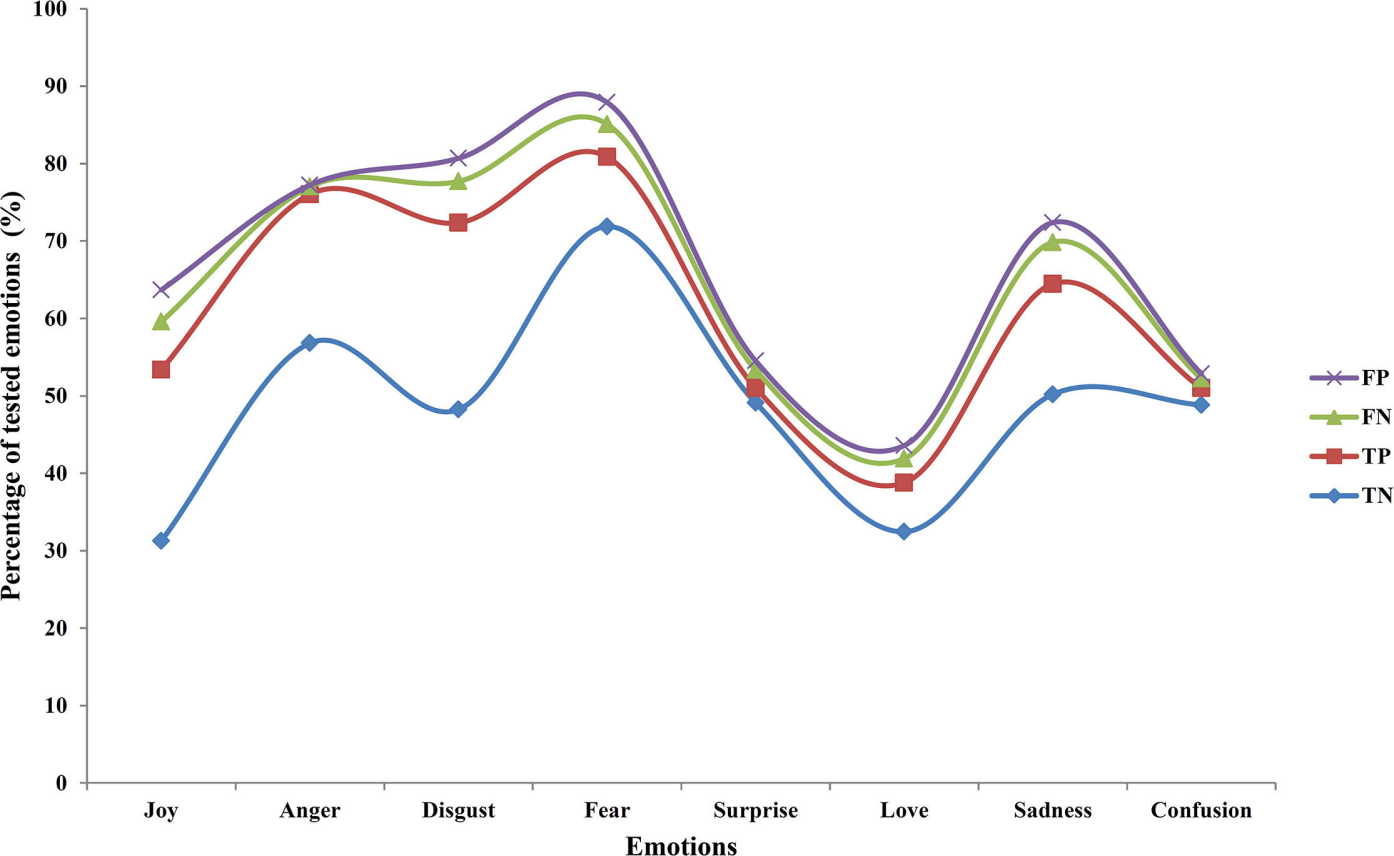
Percentage of TP, TN, FP, and FN.

To measure the accuracy of the proposed system, it has always relied on the precise calculation of the confusion matrix and mostly it is to obtain a low score over false positives (FP). Our proposed work is able to extract and identify almost 81% of true positives (TP) but it has encountered some serious challenges in detecting the false negatives (FN). This result has been vividly depicted in Table 5 that projects the amount of FP generated against FN. Figure 4 portrays the percentage of TP, true negatives (TN), FP, and FN.

**Table 5:** Calculation of the percentage of TN, FP, FN, and TP from the confusion matrix.

	**Joy**	**Anger**	**Disgust**	**Fear**	**Surprise**	**Love**	**Sadness**	**Confusion**
TN	31.27	56.81	48.24	71.88	49.11	32.45	50.19	48.82
TP	22.10	19.21	24.11	8.99	1.90	6.33	14.28	2.19
FN	6.21	1.01	5.35	4.22	2.34	3.11	5.36	1.21
FP	4.10	0.19	2.98	2.81	1.16	1.69	2.55	0.61

Eventually, each core emotion that was extracted from the tweets have been analyzed in terms of precision, recall, and f-score, and the details are provided in Table 6.

**Table 6:** Emotion classifier for each emotion extracted from COVID-19 datasets.

**Emotion**	**#Tweets**	**Precision**	**Recall**	**F-score**
Joy	273	0.781	0.689	0.735
Anger	849	0.674	0.621	0.647
Disgust	783	0.738	0.685	0.711
Fear	959	0.811	0.783	0.797
Surprise	630	0.893	0.812	0.852
Love	384	0.874	0.823	0.848
Sadness	791	0.891	0.841	0.866
Confusion	768	0.783	0.738	0.760
Macro-Average	817	0.805	0.749	0.777

**Figure 5:**
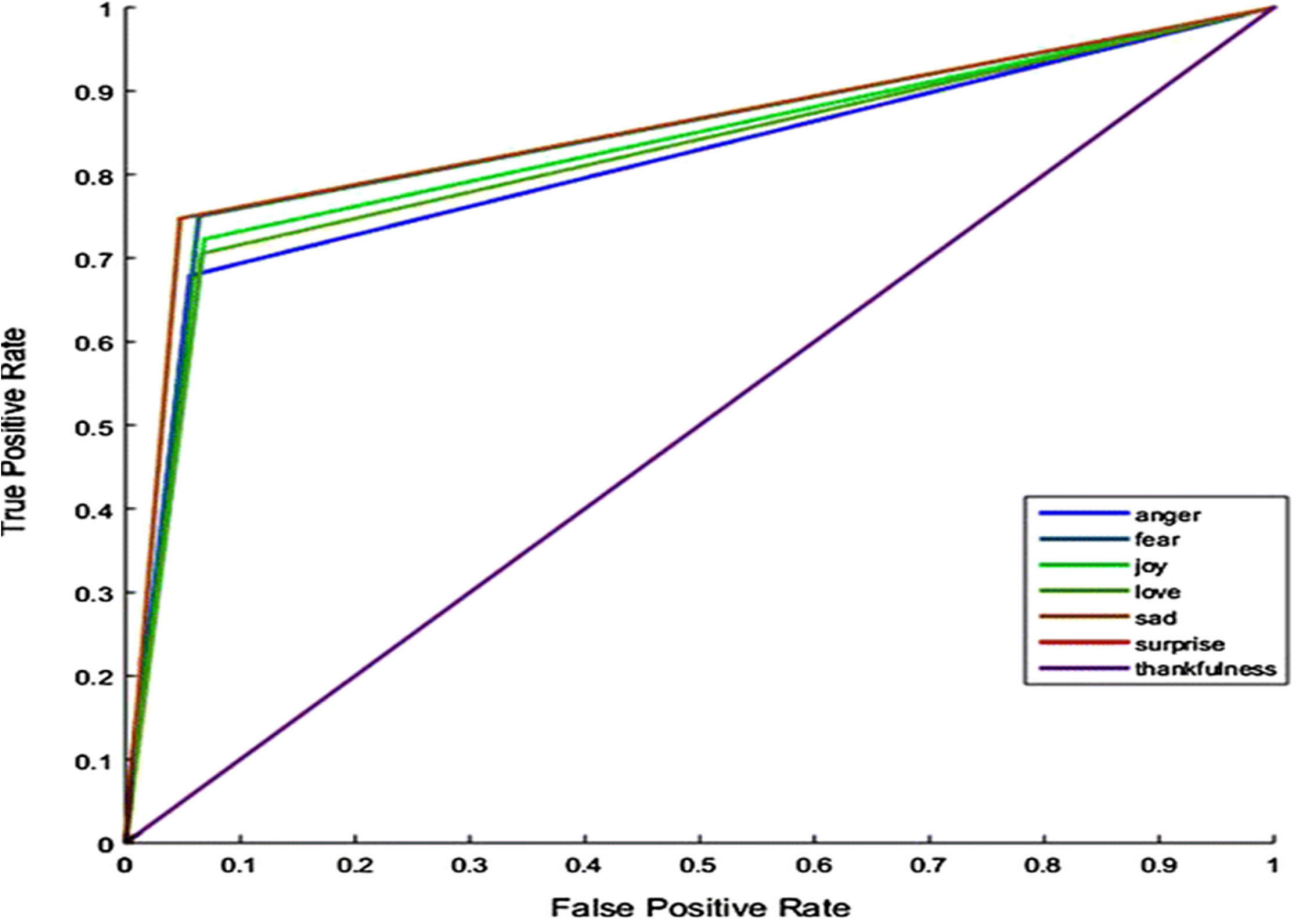
ROC curve—emotions classifier for the COVID-19 dataset.

Our proposed model considers every emotion with its associated context returned from the Emotion Ontology, and the sentiment score has been calculated based on the sum, average, and maximum count of emotion score returned by the Emotion Ontology. It has been witnessed in the analysis that our proposed system has extracted the emotional words and their associated context through our proposed Emotional Ontology. Figure 5 illustrates the receiver operating characteristic curve (ROC) of the emotion classifier for the COVID-19 dataset.

Our proposed model has been compared with some of the benchmarked baseline approaches to highlight the major differences established in terms of F1-score. Some of the limitations that have been witnessed in the existing approaches have been delineated in Table 7. Major disadvantages of the existing approaches were mostly due to the lack of contextual information, low semantic orientation between two words, and availability of limited categories to extract the emotions.

**Table 7:** Comparative analysis with existing models and their limitations.

**Reference**	**Year**	**Approach**	**Data sets**	**One sentence summary**	**Limitations**	**F1-score**
Our Proposed Model	–	Ontological Framework	COVID-19 Pandemic related Tweets	Right utilization of Ontological Framework coupled with ML algorithms such as SVM and LDA	Can work only with English Text.	0.831
Matla and Badugu (47)	2020	Machine Learning	Tweets	Deployed the Naive Bayes algorithm for classifying the Twitter messages into four emotional categories.	Absences of contextual words in the text.	0.725
Huang et al. (48)	2019	Visual and semantic attention mechanism	Tweets	Deep Multimodal Attentive Fusion for multimodal sentiment analysis of 10 million Tweets.	The fine-granularity relation between image and text pairs is not yet explored.	0.769
Ragheb et al. (49)	2019	Machine Learning	CLEF eRisk-2019 (signs of anorexia) T1 dataset	Categorized the emotions based on Attention based model.	Cannot detect the happy emotions from text.	0.710
Hasan et al. (50)	2019	Hybrid	Tweets	Deployed a supervised learning system for automatically classifying emotion in text stream messages using ANEW lexicon.	Works well only for text messages.	0.778
Almanie et al. (51)	2018	Rule Based	Tweets	Able to extract the prominent 5 emotions in a real time situation.	Low semantic orientation of textual context.	0.719
Badugu and Suhasini. (6)	2017	Rule Based	Tweets	Detected only 4 primary emotions using the Rule Based approach.	This system focused only on English sentences.	0.720

## Conclusion

The proposed Emotion Ontology model has been robust and efficient in extracting the full range of human emotions pertaining to COVID-19-related concepts. The emotions related to COVID-19 have been obtained and several emotional expressions, such as intensifiers, negator, lexical features, interjections, and conjunctions were considered for populating the Emotion Ontology. Semantic Web technologies have been utilized for creating the triples for every tweet, and the ontology construction has been manually done with the use of OWL functions. Compared to other lexicon-based emotion methods and machine learning approaches, the performance of the proposed system is satisfactory. As the tweets have been considerably informal in nature and have sparse content, in particular, the other lexical-based model and machine learning approaches failed to yield the desired sentiment score and ended up with some satisfactory precision and recall score. Our proposed study is able to extract and detect almost 81% of TP and is able to detect a considerable amount of FN. Since this study mainly focused on dealing with the English language, there is a certain limitation for extracting few emotions that exist in other languages, such as French, Spanish, etc. For future work, we will consider extracting the profound availability of emotional occupancy of resources in other languages and level their accuracy on par with standard metrics defined in the baseline approach.

## Data Availability Statement

The original contributions presented in the study are included in the article/supplementary material, further inquiries can be directed to the corresponding author/s.

## Author Contributions

SN and C-YC did the conceptualization and supervised the research. C-YC carried out the funding acquisition. SN investigated the data, performed the methodology, and implemented the software code. KS and C-YC carried out the project administration and validated the results. SN and KS wrote the manuscript. SN, KS, and SM reviewed and edited the manuscript. All authors contributed to the article and approved the submitted version.

## Funding

This research was partially funded by Intelligent Recognition Industry Service Research Center from the Featured Areas Research Center Program within the framework of the Higher Education Sprout Project by the Ministry of Education (MOE) in Taiwan and Ministry of Science and Technology in Taiwan (Grant No. MOST 109-2221-E-224-048-MY2).

## Conflict of Interest

The authors declare that the research was conducted in the absence of any commercial or financial relationships that could be construed as a potential conflict of interest.

## Publisher's Note

All claims expressed in this article are solely those of the authors and do not necessarily represent those of their affiliated organizations, or those of the publisher, the editors and the reviewers. Any product that may be evaluated in this article, or claim that may be made by its manufacturer, is not guaranteed or endorsed by the publisher.
